# Development of a Nomogram Based on Preoperative Bi-Parametric MRI and Blood Indices for the Differentiation Between Cystic-Solid Pituitary Adenoma and Craniopharyngioma

**DOI:** 10.3389/fonc.2021.709321

**Published:** 2021-07-09

**Authors:** Zhen Zhao, Dongdong Xiao, Chuansheng Nie, Hao Zhang, Xiaobing Jiang, Ali Rajab Jecha, Pengfei Yan, Hongyang Zhao

**Affiliations:** ^1^ Department of Neurosurgery, Union Hospital, Tongji Medical College, Huazhong University of Science and Technology, Wuhan, China; ^2^ Department of Geriatric Medicine, Union Hospital, Tongji Medical College, Huazhong University of Science and Technology, Wuhan, China

**Keywords:** pituitary adenoma, craniopharyngioma, radiomics, machine learning, predictive model, nomogram

## Abstract

**Background:**

Given the similarities in clinical manifestations of cystic-solid pituitary adenomas (CS-PAs) and craniopharyngiomas (CPs), this study aims to establish and validate a nomogram based on preoperative imaging features and blood indices to differentiate between CS-PAs and CPs.

**Methods:**

A departmental database was searched to identify patients who had undergone tumor resection between January 2012 and December 2020, and those diagnosed with CS-PAs or CPs by histopathology were included. Preoperative magnetic resonance imaging (MRI) features as well as blood indices were retrieved and analyzed. Radiological features were extracted from the tumor on contrast-enhanced T1 (CE-T1) weighted and T2 weighted sequences. The two independent samples *t*-test and principal component analysis (PCA) were used for feature selection, data dimension reduction, and radiomics signature building. Next, the radiomics signature was put in five classification models for exploring the best classifier with superior identification performance. Multivariate logistic regression analysis was then used to establish a radiomic-clinical model containing radiomics and hematological features, and the model was presented as a nomogram. The performance of the radiomics-clinical model was assessed by calibration curve, clinical effectiveness as well as internal validation.

**Results:**

A total of 272 patients were included in this study: 201 with CS-PAs and 71 with CPs. These patients were randomized into training set (n=182) and test set (n=90). The radiomics signature, which consisted of 18 features after dimensionality reduction, showed superior discrimination performance in 5 different classification models. The area under the curve (AUC) values of the training set and the test set obtained by the radiomics signature are 0.92 and 0.88 in the logistic regression model, 0.90 and 0.85 in the Ridge classifier, 0.88 and 0.82 in the stochastic gradient descent (SGD) classifier, 0.78 and 0.85 in the linear support vector classification (Linear SVC), 0.93 and 0.86 in the multilayers perceptron (MLP) classifier, respectively. The predictive factors of the nomogram included radiomic signature, age, WBC count, and FIB. The nomogram showed good discrimination performance (with an AUC of 0.93 in the training set and 0.90 in the test set) and good calibration. Moreover, decision curve analysis (DCA) demonstrated satisfactory clinical effectiveness of the proposed radiomic-clinical nomogram.

**Conclusions:**

A personalized nomogram containing radiomics signature and blood indices was proposed in this study. This nomogram is simple yet effective in differentiating between CS-PAs and CPs and thus can be used in routine clinical practice.

## Introduction

Pituitary adenomas (PAs) and craniopharyngiomas (CPs) are the two most common neoplasms in the sellar/parasellar region (1). PAs are benign tumors arising from the adenohypophysial cells; with an incidence of 80–90 patients per 100,000 population, they account for 15-20% of all central nervous system (CNS) tumors (2, 3). Cystic-solid pituitary adenomas (CS-PAs) refer to those PAs with such features as cystic change, necrosis, and hemorrhage. CPs are also benign neoplasms that are thought to be derived from the remnants of Rathke’s pouch or primitive craniopharyngeal duct (4, 5). CPs are relatively rare compared with PAs, with an incidence reported to be approximately 0.13-7.1 patients per 100,000 population; they account for 2-5% of all CNS tumors in adults and 5.6-13% in children (6–8). Although with different origins and pathogenesis, CS-PAs and CPs share many commonalities in their clinical manifestations, including intracranial hypertension, endocrine dysfunction, and visual disturbance. Besides, treatment considerations and prognosis are also different for the two entities. Therefore, accurate preoperative differentiation between them carries great clinical importance.

Up till now, preoperative identification of CS-PAs and CPs is primarily based on the combined information from different imaging modalities. Computed tomography (CT) is useful in demonstrating calcification, a feature that can often be observed in CPs, but this feature can also be present in some cases with CS-PAs (9, 10), thus diminishing its differentiating effectiveness. Magnetic resonance imaging (MRI), with the advantages of good tissue contrast, no bone artifacts, and multi-faceted imaging, is currently the most established imaging modality for the diagnosis of sellar/parasellar tumors (11). Several studies have investigated possible MRI features that can help differentiate between these two tumor types, such as tumor location, tumor shape, T1 image signal intensity, and cystic changes. Their results preliminarily showed the effectiveness of certain imaging features. However, a major limitation of these features lies in their subjective and qualitative nature. The actual performance of these parameters is highly subjected to the experience and expert knowledge of the neurosurgeons/neuroradiologists, which limits their clinical application. In contrast, objective and quantitative methods are preferable in these scenarios.

Radiomics is an emerging method for such tasks (12). Radiomics can extract a large number of image features in a high-throughput manner from medical images, which can quantitatively and objectively reflect tumor texture and heterogeneity (13–15). These features are usually impossible to be directly detected by the naked eye. In previous studies, radiomics has been applied to the differential diagnosis as well as prognosis prediction in various brain tumors, such as meningiomas (16–18), gliomas (19–21), and metastases (22), and lymphomas (23). However, its utility in sellar/parasellar tumors is still unclear. Besides, some preoperative blood indices, especially inflammatory markers, also deserve investigation. These indices appear to be of diagnostic and prognostic value in several neoplastic diseases including intracranial tumors (24, 25). These two categories of parameters share the advantage of being able to be retrieved directly from routine preoperative examination and thus suitable for future clinical application.

In the present study, we aimed to determine whether routine preoperative data could be used to differentiate between CS-PAs and CPs. We developed a multivariate prediction model based on a combination of preoperative bi-parametric MRI and blood indices, and internally validated its diagnostic performance. In addition, we presented the model as a nomogram for ease of clinical use.

## Patients and Methods

### Study Population

**Figure 1:**
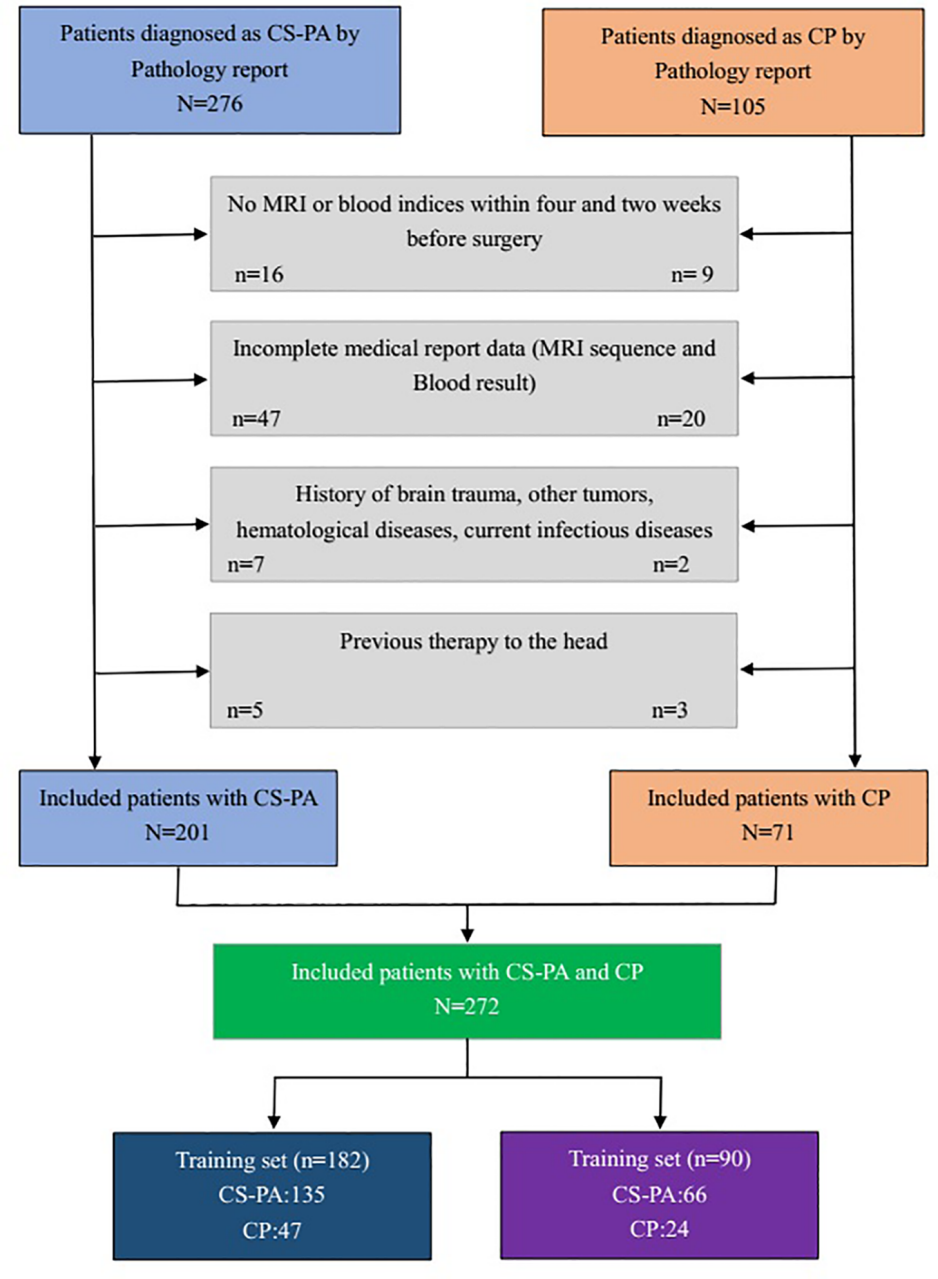
The flowchart of patient selection.

This study was conducted in accordance with the Declaration of Helsinki and approved by the institutional review board of Wuhan Union Hospital, the patients’ informed consent was waived due to the retrospective nature of the study. We collected cases from January 2012 to December 2020 that were pathologically confirmed as CS-PAs or CPs in the database. All patients were assessed by the inclusion and exclusion criteria. The inclusion criteria were as follows: (1) pathologically diagnosed with CS-PAs or CPs; (2) preoperative MRI included CE-T1 weighted and T2 weighted sequences (3) the number of lesion-bearing image slices was not less than three; (4) blood examinations included blood routine test and liver function test, which should be performed within two weeks before surgery; (5) there were no apparent signs of infection. The exclusion criteria were as follows: (1) with incomplete MRI data [for example, some patients may undergo MRI scans in other hospitals, or MRI data were incomplete/inaccessible on the Picture Archiving and Communication System (PACS)]; (2) with a history of brain trauma, brain tumors, surgery, hematological diseases, or ongoing infectious diseases; (3) have received chemotherapy, radiotherapy, or hormone therapy for any reasons before surgery. Finally, 272 patients were included in this study, of which 201 were with CS-PAs and 71 were with CPs. This selection process is presented in **Figure 1**.

### Data Acquisition and Processing

The preoperative MRI images were collected from the PACS of the Radiology Department in our hospital. These images were performed using a 1.5T (Siemens Avanto, Erlangen, Germany) or 3.0T (Siemens Trio, Erlangen, Germany) magnetic resonance clinical scanner with standard head and neck coils, and the scans were performed in coronal, sagittal, and transverse positions. The sequence parameters on the CE-T1 weighted images were as follows: The repetition time (TR)/echo time (TE)= 660/10 ms, data matrix = 256 × 256, slice thickness = 3 mm, flip angle = 90°. The sequence parameters on the T2 weighted images were as follows: TR/TE= 3000/65 ms, data matrix = 256 × 384, slice thickness = 5 mm, flip angle = 90°. The contrast-enhanced scanning was conducted within 200 s after injection of gadopentetate dimeglumine (0.1 mmol/kg). In our study, CE-T1 and T2-weighted images were used for analysis.

Region of interest (ROI), drawn manually by one researcher (Z Zhao, with 3 years of experience in PA research), was performed layer by layer on CE-T1 and T2 weighted images for all patients by using ITK-SNAP software (University of Pennsylvania, www.itksnap.org). Then, the complete 3D images of the tumor were extracted after segmentation. Lots of irrelevant information will be introduced when painting a very small tumor area. Therefore, the sections with too small tumor areas (<10 pixels) are eliminated in the process of tumor segmentation. Since the tumor region is usually not as strongly enhanced as the surrounding tissues after gadolinium-based contrast administration, CE-T1 weighted images can distinguish PAs and CPs from surrounding tissues, thereby facilitating segmentation of ROI on the images (26). In addition, CE-T1 weighted images were also referred when the tumor boundaries on T2 weighted images were uncertain.

In order to assess the stability of the identification features, 50 patients were randomly selected from the entire samples. Another experienced neurosurgeon (DD Xiao, with 6 years of experience in sellar tumor research) also described ROI on CE-T1 and T2 weighted images. Then the same feature extraction process was performed on the ROI drawn by the two researchers, and the inter-observer correlation coefficient (ICC) was calculated to evaluate the consistency of all quantitative features extracted from CE-T1 and T2 weighted images. Moreover, disagreements regarding tumor boundaries were recorded and resolved by a senior neurosurgeon (PF Yan, with 10 years of clinical experience in neurosurgery).

### Extraction of Radiomic Features

The feature extraction was conducted by using the open-source python package named pyradiomics (version 3.0.0, htps://github com/AIM-Harvard/pyradiomics) (27). The images were pre-processed before feature extraction, including normalization, discretization and resampling to a 3x3x3mm isotropic voxel size. These steps are considered to improve the reliability and robustness of radiomic analysis and are recommended by the software package developer as part of the workflow (28, 29). There are three types of features calculated in total. First-order statistic features (N=18) describe the histogram of voxel intensity values contained within the ROI through the widely used metrics, such as mean, standard deviation, and variance. Geometric features (N=14) describe the 3D shape and size of the ROI and were calculated only on the 3D mask of the ROI (i.e., independent from the gray level intensity distribution in the ROI). Textural features describing patterns or spatial distribution of voxel intensities were calculated from gray level co-occurrence matrix (GLCM, N=21), gray level size zone matrix (GLSZM, N=16), gray level run length matrix (GLRLM, N=16), neighboring gray tone difference matrix (NGTDM, N=5), gray level dependence matrix (GLDM, N= 14) texture matrices. In addition to the original image, 10 derived images were generated using LoG or Wavelet filters. Hence, a total of 1015 features were extracted for each patient: 14 shape features, 198 first-order features, and 803 textural features. In addition, the volume of the entire tumor was calculated by using PyRadiomics, too. The algorithm can be found in **Supplementary Section 1**.

### Blood Indices

The blood indices within two weeks before surgery was obtained and included from the electronic medical record system. If multiple results are available, the latest results before surgery will be used. From these results, the absolute counts of white blood cells (WBC), red blood cells (RBC), hemoglobin, platelets, neutrophils, lymphocytes, monocytes, albumin and fibrinogen (FIB) were collected. Furthermore, the following blood indices were calculated through the above indices: NLR (the neutrophil-to-lymphocyte ratio), dNLR [derived NLR, neutrophil/(leukocyte- neutrophil)], PLR (the platelet-to-lymphocyte ratio), MLR (the monocyte-to-lymphocyte ratio), LMR(the lymphocyte-to- monocyte ratio), NPR (the neutrophil-to-platelet ratio), NPI [prognostic nutritional index, albumin+(5*lymphocyte)], SII (platelet*NLR). This calculation method has been reported in many studies (25, 30, 31).

### Feature Selection Method

Firstly, the features of the CE-T1, T2 and CE-T1&T2 were normalized with z-scores in order to obtain a standard normal distribution of image intensities. Z-scores normalization is also called standard deviation standardization. The mean value of the processed data is 0 and the standard deviation is 1, the conversion formula is as follows:

$$X^{*}=\frac{X-\bar{X}}{\sigma }$$

Where X* is the transformed eigenvalue of the variable *X*, $\bar{X}$ is the mean value of the original data, σ is the standard deviation of the original data.

High-dimensional data may contain highly redundant and irrelevant information, which may lead to overfitting and greatly reduce the performance of the machine learning algorithm (32). Therefore, feature dimensionality reduction is necessary. In this study, the two steps were performed to achieve the best dimensionality reduction effect and effectively avoid overfitting. The meaningful features were selected based on the univariate statistical test (*t*-test) between the CS-PAs group and the CPs group in all patients. Then, the principal component analysis (PCA) with varimax-rotation was applied for dimensionality reduction, and in an effort to retain more variance and reduce redundancy of the variables. Furthermore, the logistic regression was conducted in all samples to compare the diagnostic performance with the feature sets of CE-T1, T2 and CE-T1&T2, respectively.

### Construction of Classification Models

Based on the above model comparison, the feature set with diagnostic performance was selected for classification model construction. Since the patient numbers of CS-PAs and CPs are quite different, the patients were divided into a training set and test set with 2:1 according to stratification cross-validation. The random up-sampling technique is used to up-sample the training set so that the positive and negative sample sizes of the training set are the same (The positive and negative samples refer to CS-PAs and CPs in this study, respectively.). The *t*-test was employed to filter the meaningless features, next the PCA was applied for data dimensionality reduction and the variance was set to 0.9. In order to avoid overfitting in the training set, the recursive feature elimination method of five-fold cross-validation was conducted to select the optimal feature set size.

Besides, for the purpose of exploring better machine-learning classification models, we applied five machine learning algorithms: logistic regression, Ridge classifier, stochastic gradient descent (SGD) classifier, linear support vector classification (Linear SVC) and multilayers perceptron (MLP) classifier. The area under the curve (AUC), accuracy, sensitivity (i.e. true positive rate) and specificity (i.e. true negative rate) were used to evaluate the predictive performance and stability of the classifiers. Then the trained model was assessed in an independent test set. The classifier with AUC>0.9 in the training set and the highest AUC value in the test set are considered to be the final radiomics model. Feature classification methods are all implemented using SCRICIT-LEARN machine-learning library.

### Development of an Individualized Nomogram

The Least absolute shrinkage and selection operator (LASSO) was performed for filtering the variables on the following clinical candidate predictors: age, gender, tumor volume, blood indices and their derivation results. A recursive feature elimination method of five-fold cross-validation was applied to select the best λ (a parameter in LASSO to be determined).

**Figure 2:**
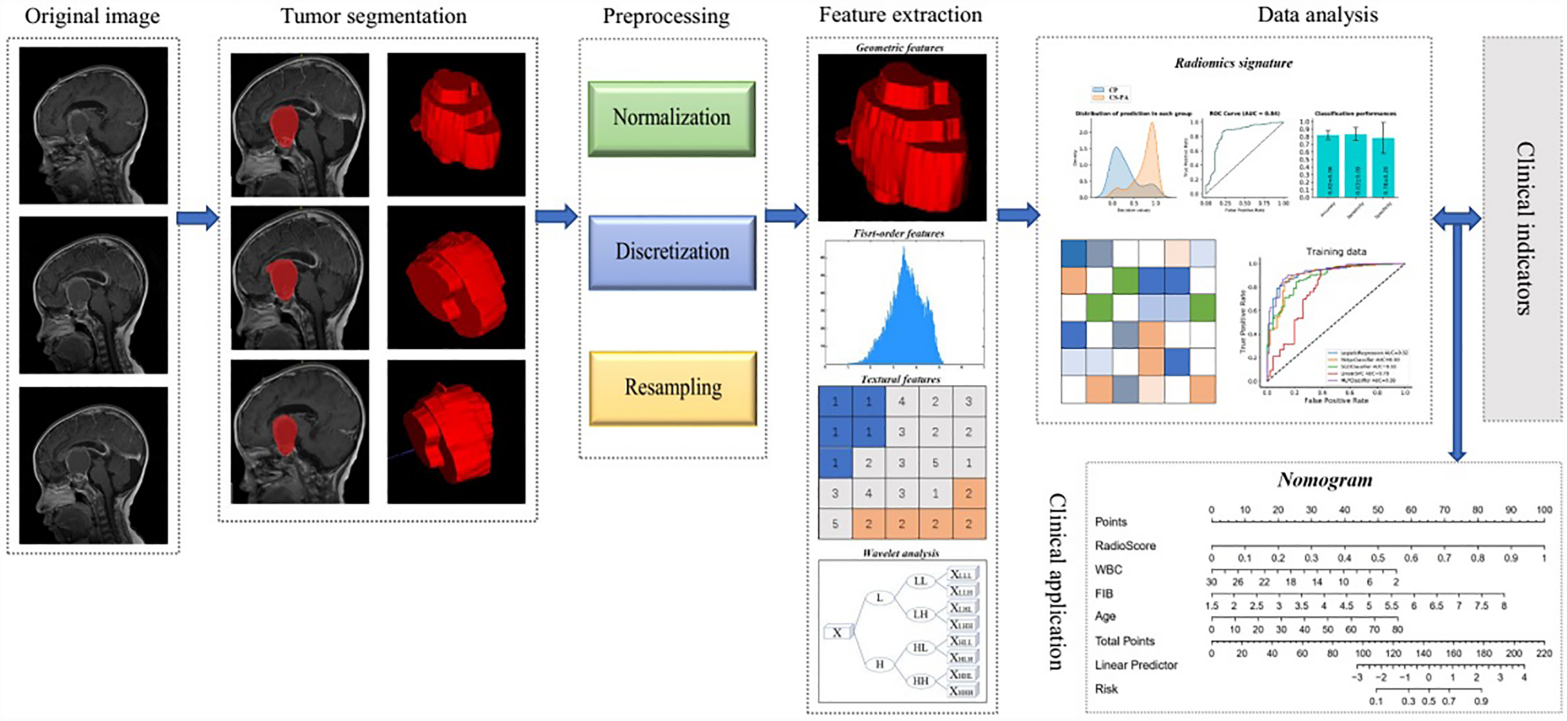
The overall workflow of radiomics processing and nomogram construction.

Giving that providing a more personalized prediction model, combined the remaining clinical parameters and the radiomics signature, a nomogram based on multiple logistic regression was established in the training set and validated in the test set. The overall workflow of radiomics processing and nomogram construction is shown in **Figure 2**.

The calibration curves were plotted for the training and test sets, and the Hosmer-Lemeshow test was conducted to assess the agreement between the predicted risks and observed outcomes. Furthermore, the decision curve analysis (DCA) was conducted to determine the clinical usefulness of the nomogram by quantifying the net benefits at different threshold probabilities.

### Statistical Analysis

The statistical analysis and figure plots were performed using R software (version 4.0.1; http://www.R-project.org) and Python software (version 3.7, http://www.python.org). The continuous variables are reported as mean ± standard deviation (SD) or median and inter quartile range (IQR), whereas categorical variables are presented as the absolute and relative frequencies. Statistical testing utilized non-parametric tests with Mann-Whitney U test and Kruskal-Wallis test for continuous variables, and the chi-square test or Fisher exact test for categorical variables. All statistical tests were two-tailed and conducted with a statistical significance level set at *p*<0.05.

## Results

### Patient Characteristics

Among of 272 patients included in this study (age, 50.11 ± 11.85 years), 201 (73.9%) were diagnosed as CS-PAs and 71 (26.1%) as CPs based on gold standard-postoperative pathological results. The patients characteristics in the groups of CS-PAs and CPs are listed in **Table 1**. Demographic results show that the patients of the CS-PAs group and the CPs group have significant statistical significance in age (*p*<0.001) and tumor volume (*p*=0.028), but there was no significant difference in gender (*p*=0.417). Among the blood indices, WBC count (*p*<0.001), neutrophil (*p*<0.001), monocyte (*p*=0.023) and FIB (*p*<0.001) are statistically different between the CS-PAs group and CPs group, and the other indicators are not statistically significant (all *p*>0.05). Moreover, satisfactory inter-observer reproducibility was achieved for both CE-T1 and T2 imaging features, with the calculated ICC range of 0.753–0.932 for CE-T1 features and 0.732–0.895 for T2 features. Therefore, it can be basically considered that the ROIs drawn by two neurosurgeons are highly consistent.

**Table 1:** Baseline characteristics of patients with CS-PAs and CPs.

	CS-PAs	CPs	P value
**N**	201	71	
**Age (mean ± SD)**	48.3 ± 13.5	37.3 ± 19.9	<0.001
**Gender (%)**			0.417
** Male**	40	102	
** Female**	31	99	
**Tumor volume (median [IQR])**	9323.26 [4949.48, 15792.47]	10828.50 [7044.23, 21169.28]	0.028
**Laboratory test (median [IQR])**			
** RBC**	4.15 [3.83, 4.46]	4.08 [3.75, 4.53]	0.996
** Hemoglobin**	126 [115, 134]	123 [114, 132]	0.474
** Platelet**	210 [172, 252]	223 [166, 276]	0.290
** WBC**	6.51 [5.16, 8.97]	9.39 [5.57, 15.10]	<0.001
** Neutrophil**	3.69 [2.66, 6.72]	6.51 [2.75, 13.42]	<0.001
** Lymphocyte**	1.73 [1.07, 2.25]	1.52 [0.70, 2.05]	0.250
** Monocyte**	0.40 [0.29, 0.52]	0.43 [0.32, 0.65]	0.023
** Albumin**	41.0 [37.4, 44.3]	41.1 [36.6, 44.4]	0.550
** FIB**	3.17 [2.78, 3.89]	3.02 [2.58, 3.37]	<0.001

SD, Standard deviation; IQR, Inter quartile range; RBC, red blood cell; WBC, white blood cell; FIB, Fibrinogen.

### Feature Selection Method

Firstly, we applied the *t*-test between the CS-PAs group and the CPs group in all patients as a prefilter for meaning features. Therefore, 730, 455, and 1185 features are retained in the CE-T1, T2, CE-T1&T2, respectively, after the *t*-test. Next, the remaining features of three feature sets were reduced by PCA and then three new matrices are formed by data information with variance greater than 0.9. It is found that the new matrix of CE-T1&T2 performed better diagnostic performance than CE-T1 or T2 by using the logistic regression model to evaluate the entire sample (**Supplementary Figure S1**). From this result, we can speculate that multi-modal MRI features are superior to single-modal MRI features in terms of differential diagnosis of tumors, which agrees with those reported by Li et al. (33).

### Construction of Classification Models

The patients were divided into the training set and test set with 2:1 according to stratification cross-validation, including 182 patients in the training set and 90 patients in the test set.

**Figure 3:**
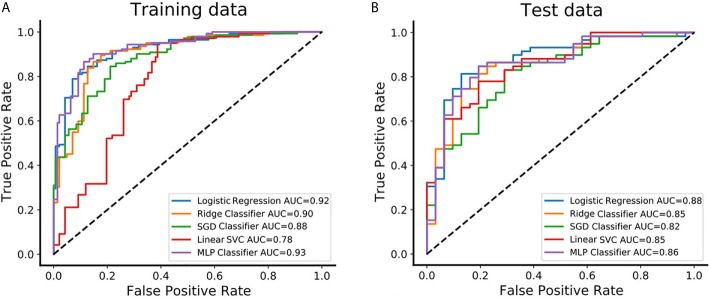
The predictive performance of distinguishing between CS-PAs and CPs in different classifiers. **(A)** The receiver operating characteristic curve (ROC) and the area under the curve (AUC) of the five different classifiers are showed in the training set, respectively. **(B)** The ROC and AUC of the five different classifiers are showed in the test set, respectively. SGD Classifier, stochastic gradient descent classifier; Linear SVC, linear support vector classification; MLP Classifier, multilayers perceptron classifier.

The feature set of CE-T1&T2 is used to construct the classification model due to predominant diagnostic performance. The *t*-test and PCA were applied for feature filtering and reduction, and features with variance greater than 0.9 were retained. Finally, an optimal feature set with 18 features is obtained through the recursive feature elimination method of five-fold cross-validation (**Supplementary Figure S2**). Based on the above representative features, they are put into 5 classifier models for training, and an independent test set is used for model verification. In the training set, the AUC value and accuracy of logistic regression are 0.92 and 0.85, Ridge classifier is 0.90 and 0.85, SGD classifier are 0.88 and 0.79, Linear SVC are 0.78 and 0.80, and MLP classifier are 0.93 and 0.87, respectively. The results in the test set are also excellent, the AUC value and accuracy of logistic regression are 0.88 and 0.83, Ridge classifier are 0.85 and 0.79, SGD classifier are 0.82 and 0.81, Linear SVC is 0.85 and 0.76, and MLP classifier is 0.86 and 0.80, respectively. These data and the 95% confidence interval (CI) of AUC are listed in **Table 2**. The receiver operating characteristic curve (ROC) of the training and test sets for five classification models are showed in **Figure 3**. The logistic regression model has represented the most reliable diagnostic performance in discrimination between CS-PAs and CPs whether in the training set or the test set.

**Table 2:** Diagnostic performance of classifiers in the training and test groups.

	Training set			Test set		
	AUC Score	95%CI	Accuracy	AUC Score	95%CI	Accuracy
**Logistic regression**	0.92	0.89-0.95	0.85	0.88	0.81-0.94	0.83
**Ridge classifier**	0.90	0.86-0.94	0.85	0.85	0.78-0.93	0.79
**SGD classifier**	0.88	0.85-0.92	0.79	0.82	0.77-0.89	0.81
**Linear SVC**	0.78	0.72-0.83	0.80	0.85	0.76-0.93	0.76
**MLP classifier**	0.93	0.89-0.96	0.87	0.86	0.78-0.91	0.80

SGD Classifier, stochastic gradient descent classifier; Linear SVC, linear support vector classification; MLP Classifier, multilayers perceptron classifier; AUC, area under the curve; CI, Confidence interval.

### Development of an Individualized Nomogram

**Figure 4:**
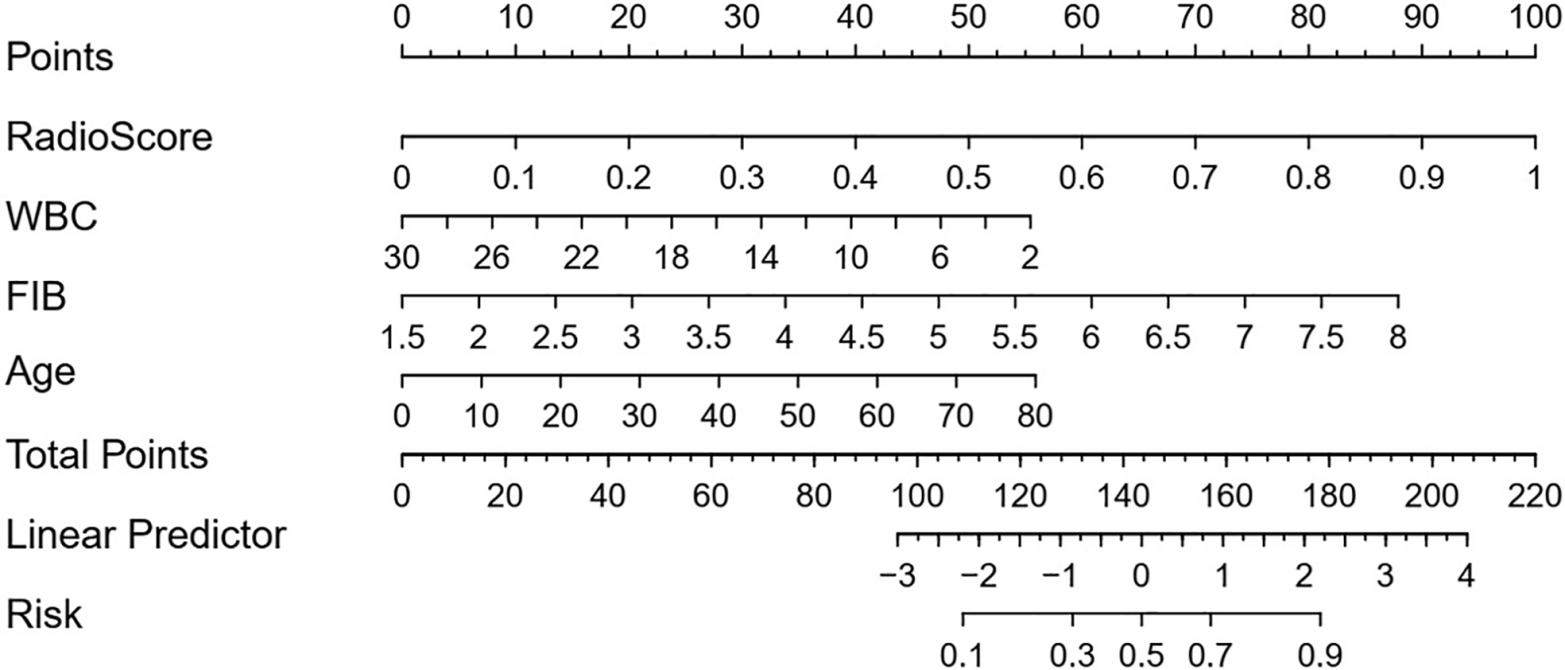
Developed radiomic-clinical nomogram. The nomogram, incorporated radiomics signature, age, WBC count and FIB, was developed in the training set. The risk represents the predictive probability of CS-PAs.

The radiomics signature, the absolute counts of WBC and FIB, and age were identified as independent factors for differentiating CS-PAs and CPs. The model that incorporated these independent predictors was developed and presented as a nomogram (**Figure 4**).

### Performance Assessment of the Nomogram

The radiomic-clinical nomogram, involved the radiomics signature, age, WBC count, and FIB, yielded an AUC of 0.93 (95% CI, 0.89–0.96) in the training set and 0.90 (95% CI, 0.85-0.95) in the test set. The radiomic-clinical nomogram was significantly superior to the radiomics model whether in the training set or the test set (*p*=0.031 and *p*=0.038 respectively; DeLong test).

**Figure 5:**
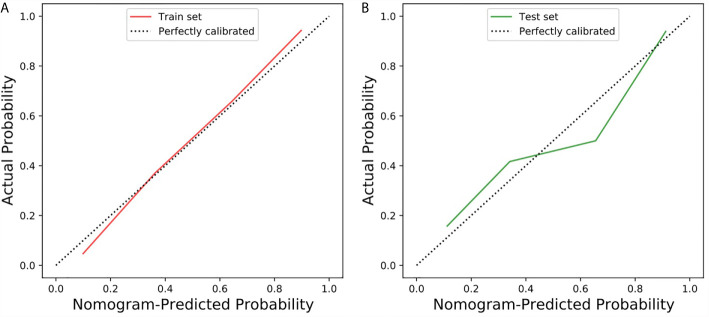
Calibration curve of the radiomic-clinical nomogram in the training and test sets. **(A)** Calibration curve of the radiomic-clinical nomogram in the training set. **(B)** Calibration curve of the radiomic-clinical nomogram in the test set. The calibration curve showed the calibration of the models in terms of the consistency between the predictive performance of CS-PAs and the actual results observed for calibration. The Y-axis represents the actual performance, and the X-axis represents the performance predicted by the nomogram. The oblique dashed line represents the perfect prediction by an ideal model. The red and green solid lines represent the performance of the nomogram in the training set and the test set, respectively. In addition, a fit closer to the diagonal dashed line indicates a better prediction. (The Hosmer–Lemeshow test showed *p*=0.367 and *p*=0.113 in the training and test set, respectively).

The calibration curve of the radiomic-clinical nomogram demonstrated good calibration in the training set and the test set (**Figures 5A, B**). The Hosmer–Lemeshow test showed a nonsignificant statistic difference in the training and test set (*p*=0.367 and *p*=0.113, respectively), suggesting no departure from the perfect fit.

**Figure 6:**
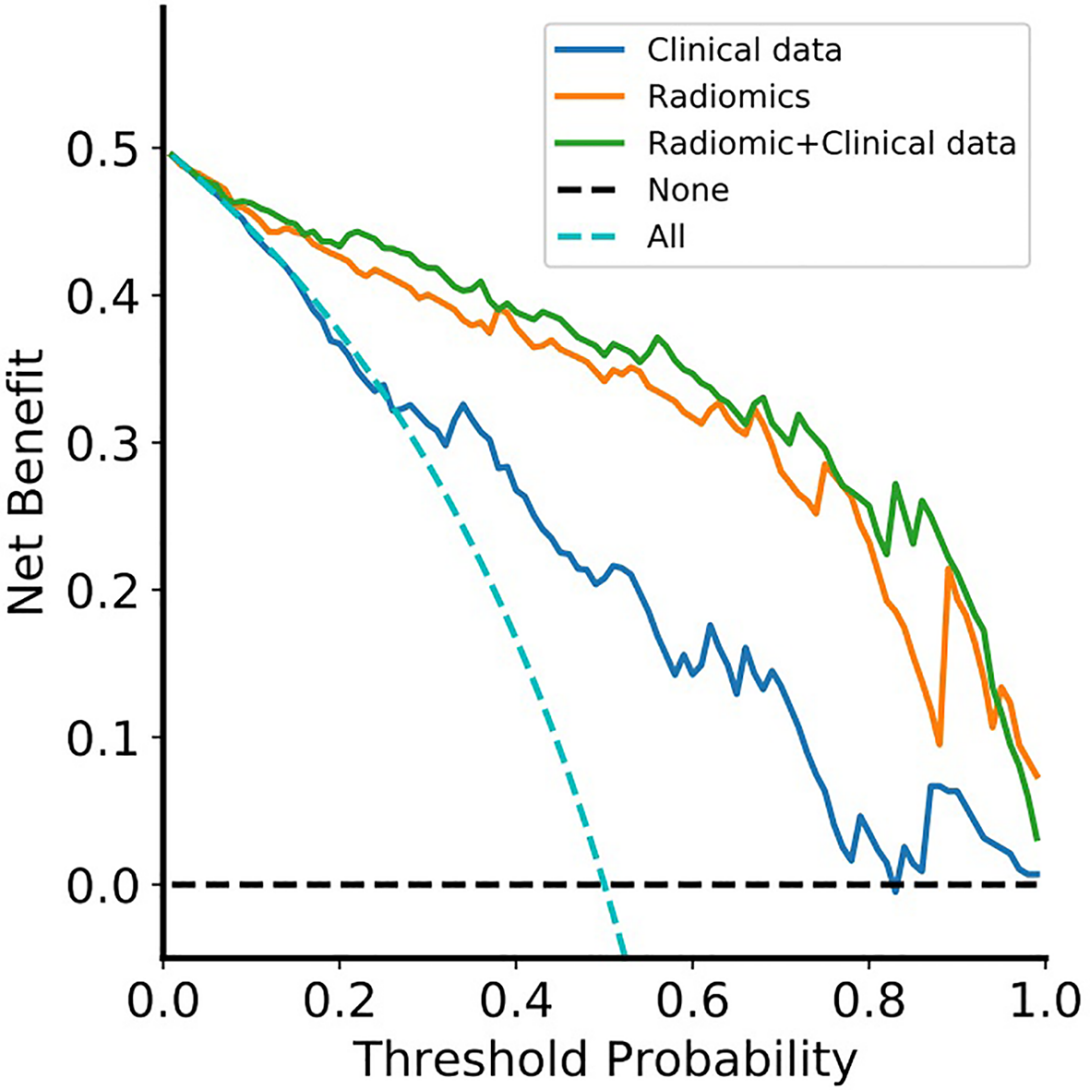
Decision curve analysis for the radiomic-clinical nomogram, radiomics model and clinical model. The decision curve showed that if the threshold probability was higher than 20%, then using the radiomic-clinical nomogram to differentially diagnose CS-PAs and CPs has a greater advantage than using a radiomics model and simple clinical model in terms of clinical application. Clinical data, clinical model; Radiomics, radiomics model; Radiomic+Clinical data, radiomic-clinical nomogram.

The DCA for the clinical model, radiomics model, and radiomic-clinical nomogram are presented in **Figure 6**. The DCA showed that if the threshold probability is higher than 20%, then using a radiomic-clinical nomogram to diagnose CS-PAs and CPs differentially has a greater advantage than using a radiomics model and simple clinical model in terms of clinical application.

## Discussion

On account of the similarity of clinical symptoms, imaging features and lesion location between CS-PAs and CPs, it is challenging to accurately differentiate between the two tumors before surgery. Existing studies have suggested that PAs and CPs are different in imaging characteristics, and the cystic change is the main criterion for distinguishing them. However, both CS-PAs and CPs will have different degrees of cystic changes, thus this criterion is of little significance in the differential diagnosis of CS-PAs and CPs. Furthermore, the treatment strategies for these two tumors are different in clinical practice. The surgical treatment is recommended for CPs once found due to the aggressive behavior, while CS-PAs can be treated by the wait-and-see approach if there are no clinical symptoms. What’s more, the surgical methods of the two tumors are not the same even if they are treated surgically. Most patients of the surgical method for CS-PAs is transnasal sphenoidal microsurgery, while CPs is basically craniotomy. Therefore, it is necessary to accurately differentiate and diagnose the two types of tumors before surgery.

Certainly, some neurosurgeons and radiologists have made sustained efforts to solve the above problems. Zhang et al. (34) constructed a model for identifying between CS-PAs and CPs based on 5 different imaging manifestations and 3 types of radiomic texture features. In the same year, their team used 5 machine learning algorithms to establish different differential diagnosis models for the two tumors based on 17 different features. The best AUC value of the training group was 0.804 and the test group was 0.845, which achieved good diagnostic results (13). Therefore, the non-invasive radiomic features based on the freely available images can be used as a more convenient biomarker for identifying these two tumors. Unfortunately, these two retrospective studies did not construct a nomogram that can be applied clinically. We have developed and verified a diagnostic nomogram based on radiomic features and blood indices, which contains four items: radiomics signature, age, WBC count and FIB. It helps to personalize diagnosis of CS-PAs and CPs before surgery by combining radiomic features and clinical risk factors into an easy-to-use nomogram.

Data processing, closely related to the performance of the model, is an indispensable process in machine learning (32). Standardization of images and data can not only uniformly transform data of different magnitudes into the same magnitude to make the data comparable, but also can improve the convergence speed and reduce the amount of calculation. Z-score, as the most commonly used data standardization method, was applied in this study. It is especially suitable when the maximum and minimum values in the data are unknown. Furthermore, the high-dimensional features of small data may lead to overfitting, and unbalanced categories of tumors may lead to misleading results (32, 35). In our study, the *t*-test was used to screen out features that are not statistically different from CS-PAs and CPs (these features have no discriminative significance), and then PCA was conducted to select sensitive component features, which could make our model more reliable and robust. The principle of PCA is to delete the redundant features (closely related variables) for all the features originally proposed, and create few new features that are pairwise uncorrelated as possible. Interestingly, these new variables keep the original information as much as possible in reflecting the information of the tumors (36, 37).

In this study, we tried to compare the classification models based on CE-T1 weighted images, T2 weighted images, and CE-T1&T2 weighted images in the identification of CS-PAs and CPs. The results demonstrated that CE-T1&T2 weighted images are better than the single CE-T1 weighted and T2 weighted images. This finding is consistent with many previous reports that the value of multi-modals imaging information is higher than that of single-modal imaging information in both diagnosis and prognosis models. The study was performed by Zhang et al. to predict the brain invasion of meningiomas. They considered that, compared with the T1-CE sequence model or T2 sequence model, the combination of the T1-CE and T2 sequences model increased the discrimination ability by 4.77% and 6.34%, respectively (38). In addition, in terms of the comparison of the single-sequence model, in the study of Zeynalova et al. (39), the result showed that T2-weighted images is better in predicting the consistency of pituitary macroadenoma. Peng et al. (35) also obtained consistent results, which showed that the T2-weighted images are better than the CE-T1 weighted images and T1 weighted images for the classification of pituitary tumor subtypes. In the preliminary model exploration of our study, T2-weighted images contains more discrimination information than CE-T1 weighted images. On the contrary, Niu et al. (40) did not think so, in the model of predicting the invasion of cavernous sinus by pituitary tumors, they concluded that the AUC value of the T1-CE radiomics model (0.796) was higher than that of the T2 radiomics model (0.720). Therefore, the feature of CE-T1 model was chosen as the final radiomics signature according to the Bayesian information criterion. The reason for this discrepancy may not be clear, as for the potential mechanism needs to be further studied.

Note that three independent clinical predictors are used for the differential diagnosis of CS-PAs and CPs, including age, WBC count and FIB. Age as an independent predictor is well understood by us. CS-PAs usually occur in young adults, while CPs occur mostly in children and adults. The age of the patients with CPs has a bimodal distribution, with the peak onset being 5-14 years old and 50-74 years old, respectively (41). White blood cells are widely existed in various tumors, especially malignant tumors, which are closely related to the important biological characteristics of tumors such as proliferation, migration, immune escape and prognosis (42–44). To the knowledge of us, malignant tumors are prone to recurrence or regrowth even after complete resection. The biological characteristics of CPs are precisely similar to this situation. Chen et al. (25) showed that in the detection results of peripheral blood inflammatory markers, the WBC and lymphocyte counts of the CPs group were higher than those of pituitary tumors, and the difference was statistically significant (*p*<0.05), which means that the progress of CPs may be related to inflammation. Furthermore, the existing reports have proved that the value of FIB in the differential diagnosis and prognosis of tumors. The theory, firstly proposed in 1865, was that the tumor is conducive to the activation of coagulation function, and then hypercoagulable state or chronic disseminated intravascular coagulation for tumor patients (45). Therefore, the radiomics model plus three readily available clinical variables make the prediction performance of the nomogram more superior.

Certainly, some limitations of this study warrant mention. Firstly, it is a retrospective study, thus some uncertain confounding factors may exist. Secondly, the patients with available preoperative MRI, blood indices and postoperative pathological results were only included for analysis, and there were relatively few samples of patients with CPs in the study population. Thirdly, all patients were from a single-center, no external validation was performed. Although we randomly divided the patients into the independent training set and test set. If a multi-center data set with different parameters is used, the performance of the model may be different. Fourthly, there are more and more multi-omics researches, thus radiomics can be combined with other omics such as genomics, so as to more accurately identify tumors and guide postoperative comprehensive treatment.

In conclusion, the study found that the logistic regression based on dual-parameters has better diagnostic performance than the other four classifiers. In addition, a new nomogram based on radiomics signature and clinical indicators was proposed, which provided a non-invasive and convenient method to individually distinguish between CS-PAs and CPs in clinical practice.

## Data Availability Statement

The raw data supporting the conclusions of this article will be made available by the authors, without undue reservation.

## Ethics Statement

The studies involving human participants were reviewed and approved by Union Hospital, Tongji Medical College, Huazhong University of Science and Technology. Written informed consent for participation was not provided by the participants’ legal guardians/next of kin because: The informed consent of patients was waived on account that was a retrospective study.

## Author Contributions

Conception and design: HYZ and PFY. Collection and assembly of the data: ZZ, DDX, CSN, XBJ, and HYZ. Language editing and grammar correction: ARJ. Development of the methodology: ZZ, DDX, and PFY. Data analysis and interpretation: All authors. Manuscript writing: All authors. All authors contributed to the article and approved the submitted version.

## Conflict of Interest

The authors declare that the research was conducted in the absence of any commercial or financial relationships that could be construed as a potential conflict of interest.
