# Supplementary material for: Development of a Nomogram Based on Preoperative Bi-Parametric MRI and Blood Indices for the Differentiation Between Cystic-Solid Pituitary Adenoma and Craniopharyngioma
Source: Front Oncol. 2021 Jul 9;11:709321. doi: 10.3389/fonc.2021.709321 (PMC8300562; doi:10.3389/fonc.2021.709321)

Supplementary Material

# Supplementary Section 1

The specific algorithm used for tumor volume calculation was as follows:

$V_{i}=\frac{O_{a_{i}}\times(O_{b_{i}}\times O_{c_{i}})}{6}$ Equation (1)

$V=\sum_{i=1}^{N_{f}} V_{i}$ Equation (2)

where, *N_f_* represents the number of faces (i.e., triangles) defining the mesh, and *V* is the volume of the mesh in millimeters cubed (mm^3^). For each face, *i*, in the mesh, defined by points *a_i_*, *b_i_*, and *c_i_*, the (signed) volume V*i* of the tetrahedron defined by that face, and the origin of the image (O) was calculated using Equation (1). The sign of the volume was determined by the sign of the norm, which must be consistently defined as either facing outward or inward of the ROI. By taking the sum of all *V_i_* values, the total volume of the ROI *V* was obtained using Equation (2). Volume calculation was conducted using PyRadiomics and Python (version Anaconda 5.0.1; Anaconda, Inc., Austin, TX).

# Supplementary Figure S1


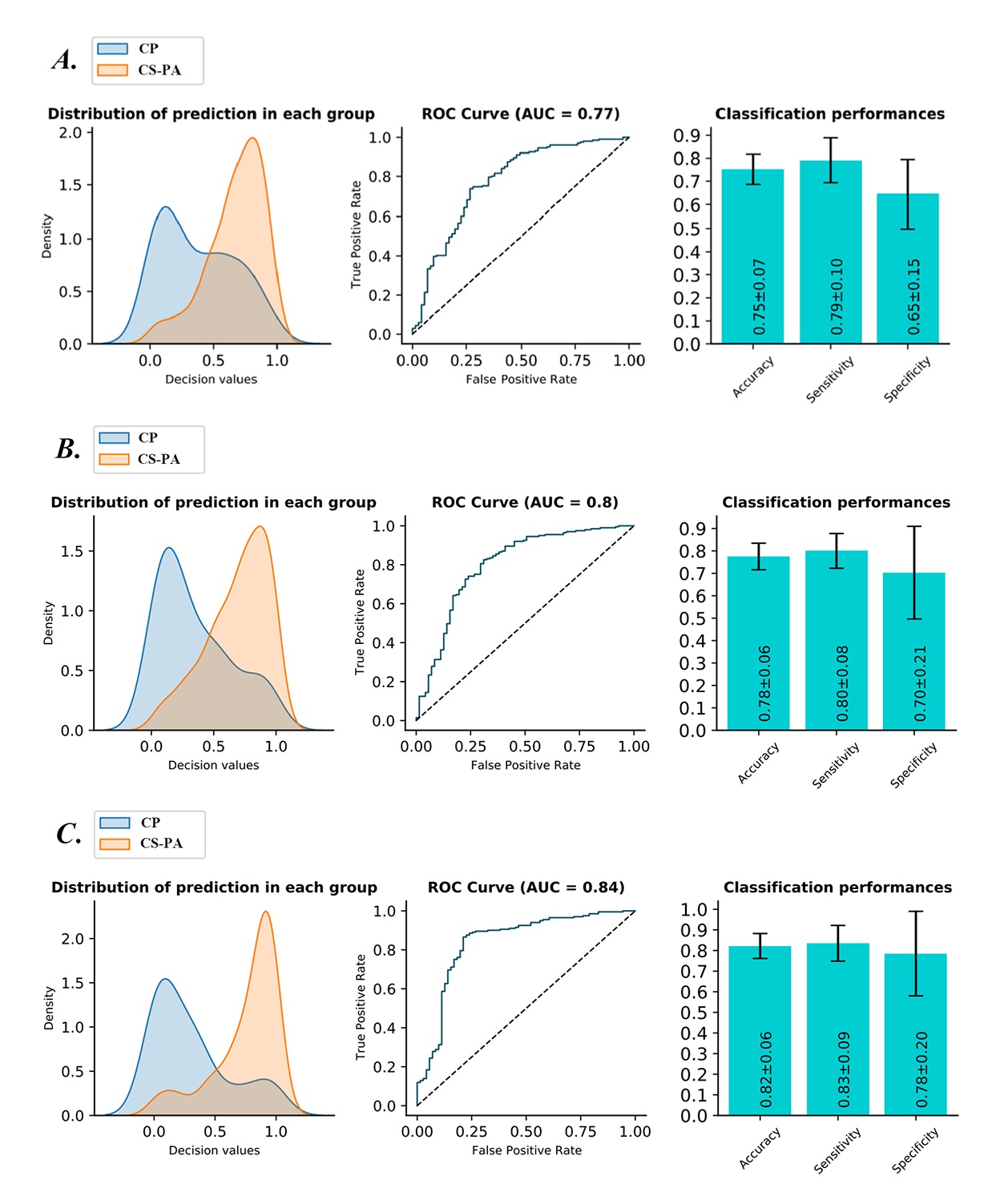
The preliminary study of classification performance on distinguishing between CS-PA and CP in different feature sets by data distribution between the two groups, AUC, accuracy, sensitivity and specificity. **A.** The preliminary predictive performance of distinguishing between CS-PA and CP on the CE-T1 feature set. **B.** The preliminary predictive performance of distinguishing between CS-PA and CP on the T2 feature set. **C.** The preliminary predictive performance of distinguishing between CS-PA and CP on the CE-T1&T2 feature set.

# Supplementary Figure S2

The optimized classification performance of distinguishing between CS-PA and CP in the most satisfactory feature set (CE-T1&T2 feature set). **A.** The optimized classification performance of distinguishing between CS-PA and CP in the training set based on the CE-T1&T2 feature set. **B.** The optimized classification performance of distinguishing between CS-PA and CP in the test set based on the CE-T1&T2 feature set.


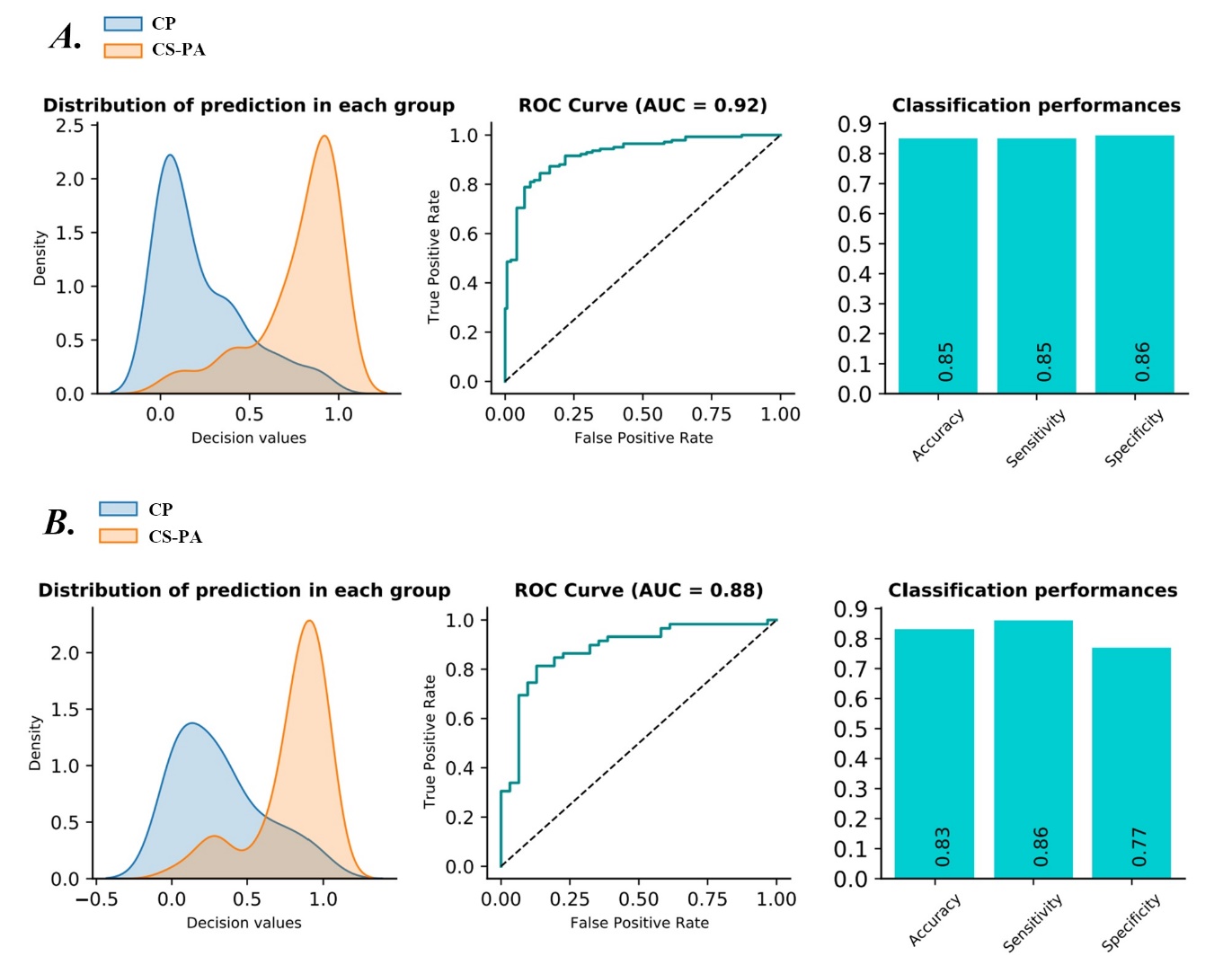

Supplement: Supplementary file 1 [file DataSheet_1.docx]
